# Patient engagement in the development of CF-CBT: A cystic fibrosis-specific cognitive-behavioral intervention for adults

**DOI:** 10.3389/fpsyg.2022.937189

**Published:** 2022-10-07

**Authors:** Deborah Friedman, Maysa M. Kaskas, Alexandra L. Quittner, Beth A. Smith, Anna M. Georgiopoulos

**Affiliations:** ^1^Department of Psychiatry, Massachusetts General Hospital, Boston, MA, United States; ^2^Behavioral Health Systems Research, Miami, FL, United States; ^3^Department of Pediatrics, University of Buffalo School of Medicine, Buffalo, NY, United States; ^4^Department of Psychiatry, University of Buffalo School of Medicine, Buffalo, NY, United States

**Keywords:** cystic fibrosis, program development, qualitative, depression, anxiety, resilience

## Abstract

Individuals with cystic fibrosis (CF) are at high risk for depression and anxiety, with negative consequences for health and quality of life. Cystic Fibrosis Foundation/European Cystic Fibrosis Society guidelines recommend routine screening, treatment, and preventative efforts. Cognitive-behavioral therapy (CBT) has a large evidence-base for depression/anxiety prevention and treatment. However, traditional CBT protocols require adaptation to address the emotional challenges of coping with CF, stressors related to disease management, and barriers to access to care. The goal of this study was to partner with the CF community to develop an innovative CBT-based intervention for the prevention and treatment of depression and anxiety tailored to CF-specific needs. In-depth feedback was collected *via* audio-recorded telephone interviews with 16 adults with CF from 3 U.S. CF centers, with purposive sampling across gender, age, ethnicity, and disease severity. A semi-structured interview guide elicited discussion of patient experiences of coping with CF, and perspectives on the acceptability of the content, structure, and delivery model of the proposed intervention. Qualitative analysis utilized a content analytic approach. Participants ranged from 21 to 53 years (*M* = 35); eight were female; three were Hispanic. Patient-reported most recent FEV1, a measure of lung function based on forced expiratory volume in in one second, ranged from 25 to 113% predicted (*M* = 72). One participant was post-double lung transplant. Qualitative interviews were analyzed thematically revealing core themes related to the experience of coping with CF. The most frequently cited CF-related stressors were Treatment Burden, Illness Uncertainty, and Financial/Insurance Stress. Participants talked about the interaction of physical symptoms and emotional distress in their daily lives, a topic not typically discussed in routine CF care. Resilience was also a major theme with participants describing strategies they use to cope with CF and hospitalizations. Description of patients’ experiences was incorporated into the program’s intervention manual and patient workbook. Participants also provided direct feedback on the proposed program. Feedback was largely positive regarding program content and structure, suggesting the acceptability of a CF-specific CBT-based intervention for adults with CF. Features to increase accessibility of care including telehealth, inpatient delivery, and team-based care were perceived as advantageous, and participants emphasized the value of a *CF*-specific mental health intervention. Qualitative findings directly informed the development of CF-CBT, a cognitive-behavioral skills-based program to promote emotional well-being for adults with CF.

## Introduction

The prevalence of depression and anxiety in people with cystic fibrosis (pwCF) is 2 to 3 times the general population (Quittner et al., 2014). Untreated depression and anxiety can have a devastating impact on health outcomes for people with this chronic, genetic illness, with negative consequences for the ability to sustain daily care, health-related quality of life (HRQoL), lung functioning, and survival (Riekert et al., 2007; Havermans et al., 2008; Smith et al., 2010; Yohannes et al., 2012; Snell et al., 2014; Schechter et al., 2021). International CF Foundation (CFF) and European CF Society (ECFS) guidelines have recommended routine screening, treatment, and preventative efforts for depression and anxiety for all pwCF ages 12 and above (Quittner, et al., 2016). Screening provides a tremendous opportunity for early intervention to prevent negative outcomes. However, access to evidence-based mental health care that addresses the needs of pwCF is limited; barriers to access include insurance or financial obstacles, the lack of understanding about CF by community-based providers, limited availability of mental health care providers with training in evidence-based interventions, and long waitlists for care (Mueller et al., 2020). A survey of 1,454 CF healthcare professionals conducted by the CFF and ECFS concluded that CF centers need additional training and educational resources to implement the mental health guidelines. Notably, 47% of providers indicated a desire for training in cognitive-behavioral therapy (CBT; Abbott et al., 2015). This speaks to the need for innovative models of mental health intervention, training, and service delivery to address the unique emotional challenges of living with CF and barriers to access to mental health care.

Cognitive-behavioral therapy has demonstrated efficacy in improving HRQoL and preventing and treating depression and anxiety in general and chronic illness populations (Butler et al., 2006; Fordham et al., 2021). There is little data on evidence-based interventions like CBT for pwCF, though emerging research in this area shows promise (Graziano et al., 2021; O'Hayer et al., 2021; Bathgate et al., 2022). Existing treatment protocols, however, require tailoring to address the specialized needs of pwCF. Trials of general CBT have typically excluded people with comorbid chronic illness conditions, and standard CBT protocols are not often easily applied to chronic illness. CBT for anxiety, for example, typically targets unrealistic fears and worries (Greer et al., 2010). However, pwCF often have rational anxiety and fears related to their long-term health and medical experiences, including worries about disease progression, disability, painful medical procedures, difficulty balancing a burdensome CF daily treatment regimen with other life goals, and a shortened life span (Ratjen et al., 2015). CBT interventions for depression include core treatment components, such as increasing behavioral activation and problem-solving. However, pwCF face unique barriers to behavioral activation and problem-solving that would not be addressed in a typical CBT protocol, such as managing CF symptoms, treatment burden, and infection control. In addition, existing CBT protocols for depression and anxiety do not include content that focuses on the impact of emotional distress on the ability to sustain daily CF care, with the potential for both immediate and downstream consequences for overall health (Cameron et al., 2022).

Our team of psychologists and psychiatrists specializing in CF mental health care and research partnered with the CF community to develop an innovative CBT-based intervention for the prevention and treatment of depression and anxiety directly addressing the needs of adults with CF. In-depth individual interviews were conducted with adults with CF across three CF centers to gather critical input to inform intervention development. A structured interview guided discussion of patient experiences of coping with CF and their perspectives on the acceptability of the content, structure, and delivery model of the proposed CBT-based intervention. Two focus groups that paralleled the structured interviews with patients were conducted with CF care teams to elicit stakeholder input from multidisciplinary providers. All of this feedback informed the design of *CF-CBT: A Cognitive-Behavioral Skills-Based Program to Promote Emotional Well-Being for Adults with Cystic Fibrosis*, a modular 8-session CBT-based intervention to prevent and treat depression and anxiety.

CF-CBT is the first mental health intervention for CF that utilized qualitative input from key stakeholders throughout the development process. It was conceptualized and designed to be incorporated within an integrated model of care, delivered by trained multidisciplinary CF health care providers, offering accessible preventive mental health care as a component of routine CF care. Integrated care models are generally preferred by patients, with strong evidence that they improve access as well as outcomes (Asarnow et al., 2015). Flexibility in mode of delivery was also incorporated into the model to improve accessibility through optional telehealth visits and the ability to conduct sessions during an inpatient admission, as needed. After the CF-CBT intervention manual, patient workbook and training program for multidisciplinary providers were developed based on CF community input, the feasibility, acceptability, and efficacy of the program were examined in a multi-center pilot (Friedman et al., 2022) and an ongoing randomized waitlist-controlled trial. Clinical trials targeted adults with mild symptoms of depression and anxiety with the goals of promoting resilience, preventing the escalation of depression and anxiety, and reducing associated morbidity.

This study presents the results of a qualitative analysis of the interviews of pwCF completed in the development of CF-CBT, using an inductive process to identify frequent themes about the experiences of coping with CF and patient perspectives on the proposed CF-specific mental health intervention. These qualitative interviews guided the CF-CBT program development with the goal of creating a mental health intervention with program resources for CF care providers and patients that is highly relevant to the needs of this population. A description of qualitative findings and how these findings guided the CF-CBT program development follows.

## Materials and methods

A semi-structured interview guide including 44 questions with follow-up probes, was developed by the study PIs (DF, AMG) with input from experts in CF mental health from each study site (ALQ, BAS). The interview guide was organized around two main themes: *Experiences of Coping with CF* and *Program Development Feedback*. Questions eliciting content about experiences of coping with CF included questions about CF-related stressors (e.g., “What do you think are the some of the top stressors that individuals with CF face?”), utilization of coping strategies (e.g., “Are there any specific strategies you use to cope with an inpatient admission?),” and experience with mental health treatment (e.g., “If you have had counseling, what do you think has been the most helpful aspect when it comes to managing the challenges of living with CF?”). Questions eliciting Program Development Feedback included the following: (1) General impressions of the program, (2) Program structure and delivery model (e.g., “Do you see any advantages or disadvantages to the program being delivered by a member of the CF team?”), and (3) session by session feedback on proposed CF-specific content (e.g., “What do you think of these examples? Do they seem relevant? Can you think of any other examples of situations that may cause anxiety in CF?”), perceived barriers (e.g., “Do you see any specific barriers to practicing relaxation for individuals with CF?”), and suggested modifications (e.g., “Do you see any ways to tailor this topic so that it can be made especially useful for individuals with CF?”).

Individual one hour audio-recorded interviews following the interview guide were conducted by phone with 16 adults with CF recruited from three U.S. CF centers between March 2017 and January 2018. Interviewers were MD and PhD investigators (DF, ALQ, and BAS). Purposive sampling was used to obtain diverse perspectives across gender, age, ethnicity, and CF disease severity. Institutional review board review determined this protocol to be exempt. Focus groups of CF care teams (pulmonologists, nurses, nutritionists, physical and respiratory therapists, and social workers) at two CF centers were also conducted. Interviews and focus group feedback informed the design of the intervention manual and patient workbook. Two of the 16 patient interviews were not recorded due to technical difficulties. In these cases, detailed hand-written notes from the interviewers were utilized. Thus, while feedback from all interviews and focus groups informed intervention development, 14 transcribed adult patient interviews were included in formal qualitative analysis in which interview text passages were categorized by theme.

Transcribed interviews were pseudonymized to preserve confidentiality and analyzed qualitatively using NVivo content-analysis software, version 12. Content-analytic methods were used to identify primary, secondary and tertiary themes based on line-by-line coding, creating a coding frame or codebook (Hsieh and Shannon, 2005). A hybrid analytic process utilized inductive reasoning to identify themes regarding the experience of coping with CF, and a mixed inductive and deductive approach to analyzing program feedback. For example, several questions elicited content regarding the deductively derived theme of “*Modifications to CBT Skill Application in CF*,” (e.g., “What do you think are barriers to staying active and social for people with CF?”), but the barriers that emerged based on that question and throughout the interviews were further coded into the subthemes of *Mental Health*, *Physical*, and *Social Considerations* in an inductive process (Bingham and Witkowsky, 2022). Consensus coding was utilized by a team of three PhD-level investigators (DF, MMK, and ALQ) to establish the major themes and codebook. Subsequently, two of the investigators (DF, MMK) met together to discuss and code the remaining 11 interviews utilizing this codebook, reaching consensus on the categorization of each text passage. The coders created codes and subcodes for each text passage until thematic saturation was reached after eight transcripts were coded, and then subsequent text passages were categorized within these themes. Participant-reported demographic information was collected during interviews and descriptively summarized.

## Results

### Participant characteristics

Patients ranged from 21 to 53 years (*M* = 35); eight were female; three identified as Hispanic/Latinx; and one was post-transplant. Of the 10 providing these data, most recent patient-reported forced expiratory volume in one second (FEV_1_), a marker of lung function in pwCF, ranged from 25 to 113% of predicted (*M* = 72). As noted above, of the 16 interviews conducted, 14 were able to be transcribed and included in the formal qualitative analysis presented here. See Table 1 depicting patient characteristics.

**Table 1:** Participant characteristics.

Age in years, mean (*SD*; range)	35.4 (12.3;21–53)
Male	8 (50.0%)
Hispanic/Latino FEV1, % predicted (patient reported) Post-transplant status	3 (18.8%) 71.5 (26.0;25–113)^*^ 1 (6.3%)
Ever diagnosed with depression/anxiety Had participated in mental health treatment (therapy/medication)	8 (50.0%) 13 (81.3%)

* Based on *N* of 10 due to missing data.

### Qualitative themes

**Figure 1:**
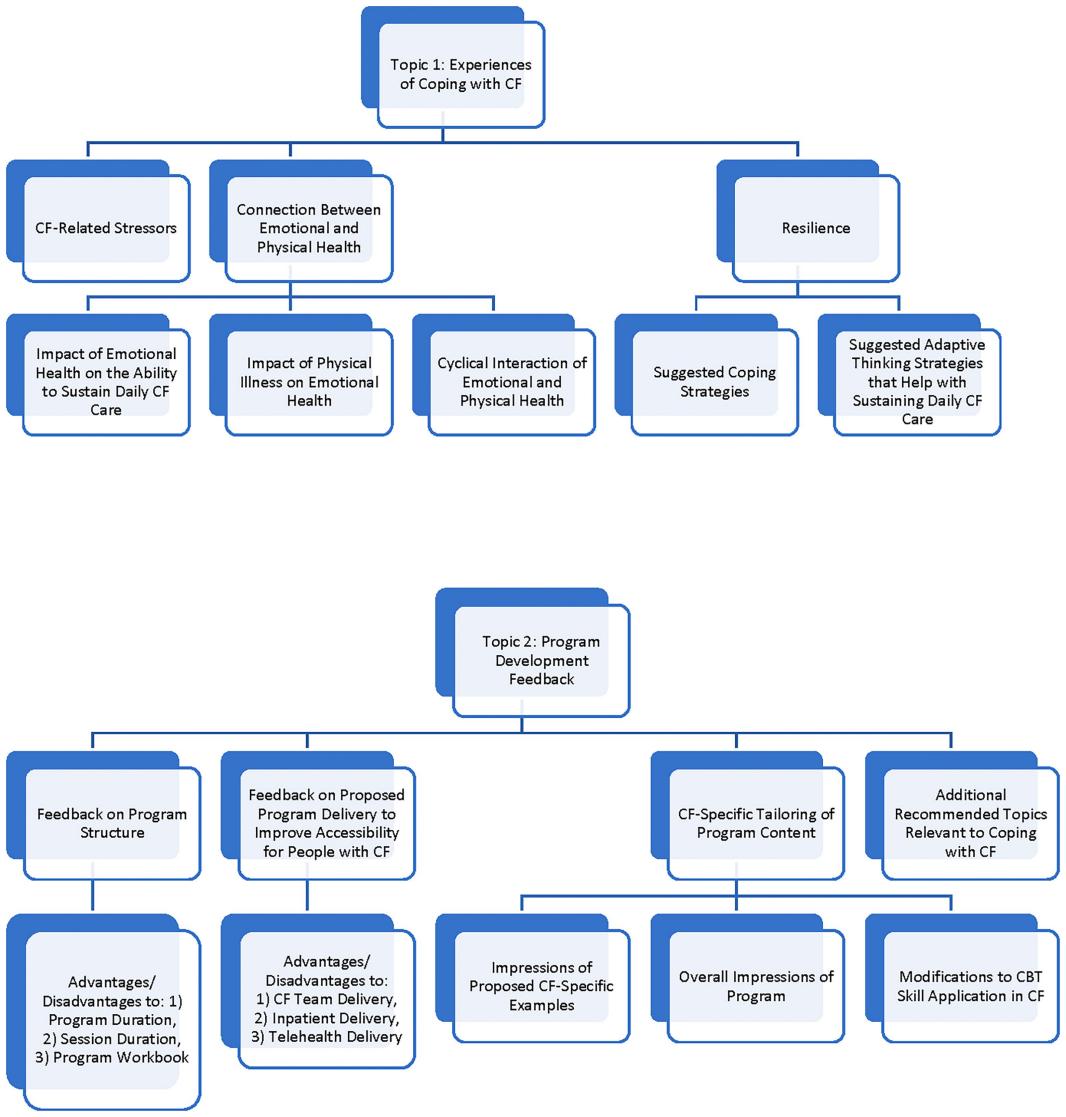
Coding frame.

There were two overarching topic areas elicited by the interview questions: (1) Experiences of Coping with CF and (2) Program Development Feedback. Figure 1 displays the coding frame that was developed in these two topic areas. Themes that were identified from the interviews within these two topic areas are presented in Tables 2–6, indicating the number of interviews in which each theme was identified, with accompanying representative quotes.

**Table 2:** *CF*-related stressors.

**Theme/Subtheme**	**Frequency**	**Exemplar quote**
Treatment burden	10	Participant 08: “Making sure you do all your treatments, you take all your medicines, you make all the appointments you have to, and you go to them, and then making sure you refill everything in time so you do not run out. It is just a constantly moving thing. It’s like you are never done with it.”
Illness uncertainty	9	Participant 08: “When you are healthy, thinking about the next time that you are going to get sick… the anticipation of being sick again is definitely getting to me, and made it hard to enjoy being healthy.”
Financial/insurance stress	8	Participant 12: “The highest payment issue in our lives is to not lose our house over my health. That was always a huge ‘keep me awake at night’ issue.”
Hospitalization	6	Participant 11: “There’s probably nothing more stressful than being hospitalized and being treated like a pin cushion.”
Self-perception/social comparison	6	Participant 06: “Comparing yourself to your peers … me and my friends, we are on the same page all the way through college … and then … you start to see, your friends have moved on. They moved out and they have got careers, some are getting married, and me, you know, I have gotten sicker.”
Disease progression	6	Participant 07: “Acquiring other illnesses kind of related from CF whether it’s severe diabetes or liver issues or bone issues.… Knowing that these things may come out as you get older, even though you are taking good care of yourself is scary.”
Fear of death/dying	6	Participant 10: “When I was growing up, they always had a number, a life expectancy number, and it was 8, when I was born, you know, you are not going to live past grade school and then you get past grade school and the number is like 15 and then it’s 18 and then it’s, 24, then 30. Growing up, you are always trying to get above the age.”

**Table 3:** Connection between emotional and physical health.

Theme/Subtheme	Frequency	Exemplar quote
**Impact of emotional health on the ability to sustain daily CF care**		
Low motivation	4	Participant 02:“I also notice … when my mood is not as good, it’s harder to – I cannot say I’m ever enthusiastic about treatment, breathing treatments for my airway clearance, but I feel less motivated … I’m feeling lousy anyway so what does it really matter if I miss a treatment … those thoughts creep in more easily.”
Negative thoughts that impact adherence	9	Participant 08: “Sometimes when I’m not feeling well, that’s really overwhelming … so sometimes I think, if I cannot do all of them at once, then I just will not do them.”
**Impact of physical illness on emotional health**		
Loss of control	5	Participant 14: “If I wake up, let us say, coughing a lot, shortness of breath, I already know it has to do with CF – it does get me mad because I do not understand how I’m feeling [awful] that day – if I’m doing everything I’m supposed to do.”
Mood, anxiety, and depression	7	Participant 02: “Sometimes the first thing I notice [when I’m having an exacerbation] is that my mood is not as good, you know, that I’m maybe a little bit more irritable, that I become impatient or things annoy me that might not typically even bother me… So I think it’s very tied together, for me anyway.”
Negative thoughts	7	Participant 04: [I think] “I do not have any impact on my health anyway.”
*Self-critical thoughts*	5	Participant 04: [I think] “I’m not good enough. I’m not able to do the things I want to do. I’m not contributing to my family like I should. I’m not worthy.”
*Social comparison*	7	Participant 13: “I also am wondering, at work when I’m coughing – what do people think? And even like if I’m in a breakroom, I kinda step aside and take my medicine in the bathroom because I do not want people to see me. My mind wonders what people are thinking – and if they think I’m weird … a big thing is doing treatments around other people. Unless it’s a close family or friend, I’m having a hard time learning how to do that around others.”
**Cyclical interaction of emotional and physical health**		
Description of cycle	7	Participant 09: “They are 100% in every single way possible to the highest degree tied…You immediately start to see the integration of your mental health when your body starts to feel awful…because you just become so weak and unable to do all the things you want to do, and that has a serious effect on your mental health. Likewise … I know myself and I know a bunch of other CF’ers too, when they start to develop or go through life stressors or anxiety flares up or depression is bad, that may just be a mental thing, but next thing you know, it affects their physical ability to take care of themselves or to keep eating correctly, or frankly to really [care] that they need to do what they need to do on a daily basis. And it severely impacts your physical health because …. you just start to get sick…. So, [physical and emotional health] go hand in hand in every way possible.”

**Table 4:** Resilience.

**Theme/Subtheme**	**Frequency**	**Exemplar quote**
Selected suggested adaptive thinking strategies that help with sustaining daily CF care*		
Self-Compassion/Do not be too hard on yourself	6	Participant 08: “I would have to tell myself, I’m not feeling well and I need to take care of myself and I’m not in a rush. There’s no reason really that I have to do all these things at once because I’m not going to go anywhere today. I’m going to stay home and not feel well, so I can just sit on the floor with my vest all set and do a nebulizer first, and then do the vest and I can shut it off as many times as I need, to take a break, and then I can do the other nebulizer afterwards or later if I’m feeling really bad. I think it’s good that I conditioned myself to do these treatments consistently in a certain way, but then it was also good to realize it’s okay if I restructure the way that I normally do things to adapt to how I’m feeling.”
Identifying your personal goals and values	3	Participant 11: “Finding yourself good goals, knowing what you want to do, what you want to feel like, what you want to look like.”
Selected suggested coping strategies: coping with hospitalization*		
Distracting, fun activities	10	Participant 01: “What do you do at home to make yourself feel better? Do you have a cup of tea and listen to music? Do you read a book? Do you read a magazine?”
Stay active	8	Participant 06: “Physical activity. I try to do it right away; getting out of the room helps.”
Social support	7	Participant 04: “I normally reach out to people and make sure I’m in contact with friends and family.”
Acceptance	3	Participant 08: “The main thing I had to do was just accept that I was there and no amount of not wanting to be there was going to change that, and then keep myself busy with little things*…* I have my computer, I have games on it. I bring crossword puzzles to focus my brain on something*…* I would look forward to the food tray coming*…* anticipating that*…* I would not say making the most, but - making a better time out of little things.”

* Categories were included in the table when mentioned by 3 or more participants.

**Table 5:** Increasing accessibility/delivery considerations.

Theme/Subtheme	Frequency	Exemplar quotes
**CF Team Delivery**		
Advantages	13	Participant 01: “If … CBT is part of the overall cystic fibrosis care… it’s letting the patients know that CBT is real and it works, and that there’s a lot of valuable lessons [in CBT].” Participant 06: “The CF team knows us very well … and it’s their specialty.” Participant 11: “They’re already familiar with the disease so you do not have to explain to them what [CF] is.”
Disadvantages	8	Participant 02: “If there was something that you were not comfortable discussing or … if you wanted to kind of compartmentalize it and to keep that separate.” Participant 04: “It may be hard to be honest with a member of the team if you are trying to say you are compliant but you are really not.”
**Inpatient Delivery**		
Advantages	13	Participant 01: “Continuity is important.” Participant 03: “When I’m inpatient…it’s lonely and you have a lot of time with yourself … sometimes that’s a little rough because I’m hardest on myself and sometimes I’m not my own best friend.” Participant 07: “You might get more even heightened fear and anxiety when you are in the hospital … so it could be really beneficial.”
Disadvantages	1	Participant 14: “From experience, I’d rather just concentrate on getting better. And once I feel better, I can…have an appointment.”
**Telephone Delivery**		
Advantages	10	Participant 04: “Having it accessible via phone would make the follow-through better because you’d get more [people] sticking with the program.” Participant 09: “Definitely an advantage…for some people including myself who live a little bit further from the hospital. They [may] have…long work hours, [so phone delivery is] more flexible.”
Disadvantages	4	Participant 02: “Personally, I like eye contact and…feel a little bit more comfortable in person.” Participant 11: “There’s a lot of body language you’d be missing out on.”

**Table 6:** CF-specific tailoring of program content.

**Overall Impressions of Program**		
Exemplar quotes		Participant 01: “It’s an important component that’s missing from overall care.”
		Participant 02: “I have used CBT in the past, but my experience was that it was difficult to access…. To say that it changed my life would be an understatement…. It was so helpful, so valuable…. In the end, I really felt different.”
		Participant 08: “One of the most helpful things was receiving the validation that this was a significant problem I was having, because I had never gone for mental health treatment before. I was always so focused on physical health.… This is a valid issue that lots of people struggle with, and I’m not just being a crazy person because I feel like I am having these problems. They are legitimate, and there are ways to treat them and take care of them, just like there are ways to treat cystic fibrosis.”
**Suggested Modifications to CBT Skill Application in CF**		
**Relaxation**		
**Theme/Subtheme**	**Frequency**	**Exemplar quote**
Physical considerations	8	Participant 08: “If I’m having an exacerbation and not feeling well, trying to breathe deeply makes it worse and makes me cough a lot.”
*Suggested Modification*	7	Participant 09: “Some of us can take huge breaths … but [for] others … guided imagery that does not require a lot of physical effort might be infinitely better and a little bit more tailored.”
**Behavioral activation**		
**Theme/Subtheme**	**Frequency**	**Exemplar quote**
Mental health considerations	4	Participant 03: “It’s so much easier to say oh, let us just cancel it…. It’s so much easier to just say that than to push yourself and try.”
*Suggested Modification*	3	Participant 08: “There does not have to be a specific concrete goal that you need to reach and you are not failing if you do not reach a certain level … you are just accomplishing what you are able to at that time, which is better than doing nothing.”
Physical considerations	14	Participant 05: “Well, it goes back to … the physical limitations. I have a hard time when I’m not using oxygen, being able to maintain a conversation for extended periods of time. So like, you know, I have to do – even just doin’ stuff like, you know, making sure I have planned out what I’m gonna say, so that I’m not saying too much. So I do not run out of breath.”
*Suggested Modification*	3	Participant 04: “Think about what’s realistic … a half hour activity is good as opposed to a 3-h activity.”
Social considerations	7	Participant 01: “I have a fear of being around too many people and in public areas and being exposed to germs.”
*Suggested Modification*	4	Participant 02: “I know when I’m not feeling as well … I do not tend to reach out to friends so … if I’m in regular contact with people, say, ‘if you guys do not hear from me for a week, could you just send me a text and say hey, how you doing’ or have friends check in.”

#### Topic 1: Experiences of coping with CF

Coding of the themes related to Experiences of Coping with CF identified three major themes: *CF-related Stressors*, *Connections between Emotional and Physical Health*, and *Resilience*.

Participants were asked directly about their “top CF-related stressors,” and themes related to CF stressors emerged throughout the interview when discussing how best to address these concerns in the proposed CBT-based intervention. The most frequently cited CF-related stressors were: (1) *Treatment Burden* (i.e., the amount of time and energy required for daily CF self-care), (2) *Illness Uncertainty* (i.e., difficulty planning or setting life goals due to unpredictability of exacerbations, need for hospitalization, or disease progression), and (3) *Financial/Insurance Stress* (i.e., concerns about paying for treatments and hospitalizations). See Table 2 for exemplar quotes. One participant described *Treatment Burden* as follows:

Participant 04: *“The overall burden of managing the illness with medications and the time that takes, and I think the inability to really fully engage in life like you would like to.”*

Illness uncertainty, a common component of chronic illness conditions, refers to the experience of unpredictability in relation to symptom presentation and long-term course. As noted, in CF, there is a lack of predictability in relation to illness exacerbations, need for hospitalization and disease progression. The following are several additional illustrative quotes describing the theme of *Illness Uncertainty*:

Participant 03: "*It’s hard to plan for the future. It’s hard to make long-term plans"*

Participant 04: *"I think with …not being able to plan ahead, like with school, with work, are you able to meet the demands of that situation, like am I going to be have to be out of work, am I going to be sick and not be able to be there."*

Participant 04: *"And I think, … am I able to – am I going to be able to have a so-called normal life, you know, get married, have kids, contribute to society, have a job*."

Additional CF-related stressors that were mentioned in at least six interviews were: *Hospitalizations*, *Self-Perception/Social Comparison*, *Disease Progression*, and *Fear of Death/Dying*. However, many other CF-related stressors were identified in five or fewer interviews, such as *Clinic Appointments and Medical Procedures*, *Communication and Disclosure about CF*, *Survivor Guilt*, *Dating and Relationships*, and *Family Planning*. In total, a list of 18 CF-related stressors were ultimately included in the initial CF-CBT session focused on increasing awareness of one’s response to stress, linking attitudes and actions in response to stress and well-being, and identifying coping strengths. The rationale for including a long list of CF-related stressors that were identified by study participants was to normalize these experiences and decrease the stigma related to discussing these concerns.

##### Connection between emotional and physical health

Participants were asked whether they saw a connection between emotional and physical health in their own lives, and this mind–body link was a major theme mentioned throughout the interviews. Three subthemes were identified. The first was *the Impact of Emotional Health on the Ability to Sustain Daily CF Care*. Exemplar quotes illustrating this theme are included in Table 3. Participants described how low mood significantly affected their motivation to complete their daily treatments. Not surprisingly, negative thinking patterns that affect motivation were described as more prevalent during periods of low mood. Negative thought patterns often involved “all-or-nothing” thinking, for example, believing that if it was not possible to do 100% of treatments, it was not worth doing any treatments. Other participants noted that negative thoughts about how they would be viewed by others interfered with doing treatments. For example, one participant stated:

Participant 01: *"…it's embarrassing…I mean I can’t imagine having been in college and having to break out my neb or a vest.”*

Another subtheme was the *Impact of Physical Illness on Emotional Health* (Table 3). Participants described the impact of physical illness on their mood, noting increased symptoms of anxiety and depression during times of CF symptom exacerbation. Many participants talked about feeling a *Loss of Control* when they were experiencing an exacerbation of symptoms or a need for hospitalization, despite their best efforts to maintain their health. Negative thinking patterns emerged as a prevalent subtheme, with participants describing overgeneralized patterns of thinking that nothing they do affects their health status; self-critical thoughts that they should be doing better or be able to contribute more to their families or to society; and negative thoughts comparing themselves to peers or worrying about being judged negatively by others for CF-related symptoms, disability, or treatment requirements. Exemplar quotes are included in Table 3. An additional example follows:

Participant 12: *"it was something I always could sort of control and maintain and stay on top of…Like, I could do my therapy and then I could feel pretty good after that… It was probably when things got out of hand with me that I couldn’t control anymore – that I just knew, I was on the losing end of it and I just didn’t know what the next day or next week would bring upon me.”*

Finally, participants described a *Cyclical Interaction of Emotional and Physical Health*. In different ways, they described how physical illness and emotional health are intimately tied together. It can be difficult to tease apart whether the cycle begins with increased stress and low mood or with decreased physical well-being, and there is an interactive effect in which each symptom complex (emotional or physical) intensifies and maintains the other. See Table 3 for one participant description of this cycle. Additional quotes exemplifying the cyclical interaction between physical and emotional health follow:

Participant 03: *“The worse you feel, you want to do less, but then the less you do, the [worse] you feel. It’s a cycle.”*

Participant 06: *“*(*Emotional and physical health are*) *very related … Depression can come at any time and can make you feel physically sick. I feel really fatigued and I don’t want to do anything, and then the next day I feel fine, so what was going on? Even when you are feeling physically well, it can weigh you down.”*

Participant 07: *“If you don’t feel well physically, you may… think more negatively and that can have a dire effect long term… In a way, your mental health may be even more important than your physical health.”*

##### Resilience

Resilience was a third major theme in the topic area of Experience of Coping with CF, in which participants described strategies they use to cope with CF on a daily basis and more specifically, to help with sustaining daily CF care and inpatient admissions for CF exacerbations (Table 4). At least three participants endorsed the following strategies for coping with CF-related stress: *Relaxation, Meditation, or Mindfulness; Physical Activity or Exercise; Distraction; Identifying Sources of Anxiety and Breaking these Down into Manageable Parts; Practice Disclosing about CF to Others; Reframing Negative Thoughts and Positive Self-talk; and Use of Social Support*. Fewer than three participants shared that they managed CF-related stress by *Preparing in Advance for Admissions; Focusing on Structure or Routine; or Focusing on Rest*.

When asked about what helps pwCF to stay motivated to sustain daily CF care, participants identified several types of adaptive thinking patterns which align well with a CBT-based approach to stress management and were included in the intervention manual. The most often cited thinking patterns were coded as *Self-Compassion* (“Do not be too hard on yourself”) and *Identify Your Personal Goals/Values*. For example, regarding *Self-Compassion*, one participant (01) stated *“[I think:] I did not do my best work today, but I have another shot at it tomorrow,*” and another (Participant 13) stated “*Do not punish yourself if you fall back*.” At least two participants also identified the strategies *Do not Overthink* and *Make CF Treatments a Routine* as helpful in staying motivated to complete daily CF treatments.

Participants were also asked about strategies they used to cope with being in the hospital for treatment of a CF exacerbation. Frequently cited strategies were coded into the following categories: *Distraction/Fun Activities*, *Stay Active/Engage in Physical Therapy*, and *Social Support* (e.g., reaching out to friends and family, engaging with hospital staff whom they know), and *Acceptance*. The category of Acceptance can be described as letting go of the fight to stay out of the hospital, which may have been adaptive up to that point, and instead focusing on the benefits of being treated and making the best of the current admission.

#### Topic 2: Program development feedback

In addition to seeking input on coping experiences, participants were asked in-depth questions about the acceptability of the proposed CBT-based program structure, mode of delivery, and content. The four major themes that emerged were: (1) *Feedback on Program Structure*, (2) *Feedback on Proposed Program Delivery to Improve Accessibility*, (3) *CF-specific Tailoring of Program Content, and* (4) *Additional Recommended Topics*.

##### Feedback on program structure

Participants were given information on the proposed program, beginning with a description of the rationale for a CF-specific intervention and the benefits of CBT. Participants were informed that the proposed program consisted of eight individual, weekly sessions of CBT, each session lasting approximately 30 minutes and facilitated by an existing member of the patient’s CF-care team who would receive specific training in the program. Participants were also told that each session would be guided by a workbook that included more information and opportunities for skill practice. Responses to the proposed length of the program (eight sessions) were mixed, with nine of fourteen participants describing it as reasonable, two describing it as too short, one describing it as too long, and the remaining two participants believing that program length should be dependent on individual need. For example, one participant mentioned that some people might require more sessions for skill practice:

Participant 09: *“I’d [recommend] a little more flexibility, maybe [for] somebody who is having a hard time developing CBT skills.”*

Flexibility was also recommended by 11 of 14 participants regarding program length, with several recommending a range of 45–60 minutes rather than the 30 minutes initially proposed. However, nine of fourteen participants described 30 minutes as reasonable, with Participant 06 noting that some pwCF *“can get tired from talking for longer.”* Participants were also asked about their impressions of a patient workbook that covers the core topics. Thirteen of fourteen participants described advantages to the workbook, including the ability to take notes, use the workbook for reference and to guide practice between sessions. For example, one participant stated:

Participant 05: *“Retention is always based on repetition…being able to reinforce [what you learn] is good if it ties into the workbook.”*

One participant shared a potential disadvantage of the workbook, noting that some pwCF might have challenges with reading comprehension and miss important content.

##### Feedback on proposed program delivery to improve accessibility for people with CF

The intervention was developed to facilitate greater access to evidence-based care for pwCF. These included (1) integration into routine CF care through delivery of the intervention by trained multidisciplinary CF care team members, (2) use of telehealth, and (3) inpatient delivery for those who were medically hospitalized to maintain to maintain continuity of care. For each of these domains, the following question was posed, “What do you think are the advantages and disadvantages?” to this specific aspect of the model (Table 5). CF team-based care integration was largely viewed as a beneficial component to the proposed intervention model. Advantages of CF care team delivery were discussed in thirteen of fourteen interviews and included statements about the benefits of familiarity and comfort with CF care team members, the provider’s knowledge of CF, and easier access to care. For example, one participant stated:

Participant 02: *“Advantages, I think, are that it’s very easy to access and you don’t have to bring another care provider in, get to know somebody and build a level of comfort with them”*

Eight participants also noted possible disadvantages to an integrated delivery model, with six citing privacy/confidentiality and role conflict possibly limiting a patient’s ability to be as open as they might otherwise be with a provider who is not also a member of their CF care team. For example, the same participant who cited the advantage above also stated:

Participant 02: “*The only disadvantage I can really think of was if, you know, I know that in our clinic staff pretty much know everything about our lives anyway, but if there was something that you weren’t comfortable discussing or that you wanted to have somebody sort of out of that loop, that would be the only drawback that I could think of, if you wanted to kind of compartmentalize it and to keep that separate.”*

One participant noted that CF care team delivery may increase the burden on the CF care team and another noted concern about the level of training or expertise of non-mental health CF providers delivering this intervention.

Numerous advantages to telehealth delivery were cited, such as decreasing the burden of travel to the hospital, and not having to travel or cancel appointments when sick or due to weather. One participant stated:

Participant 13:*“I think it’s a great advantage because I have a hard time now getting in therapy sessions with my work schedule, so even to be able to set a time on a lunch break, to be able to talk, I think that’s a great idea.”*

Participants also noted that they may feel more open to talk about their concerns in their home environment. The few disadvantages to telehealth delivery included wanting to meet with the provider in person, at least initially, or feeling more comfortable in-person, and two participants noted that there may be difficulty finding a quiet, private space for a session at home. The CF-CBT intervention model was originally proposed to include an initial in-person visit followed by optional in-person or telephone visits. However, during the COVID-19 pandemic, the study team pivoted to telehealth visits for all eight sessions.

The option of inpatient delivery was also seen as positive by participants, with thirteen of fourteen interviews mentioning the benefit to continuity of care, access to a stress-management intervention during a particularly stressful time, and the ability to target the specific types of stressful experiences that arise during an inpatient admission. One participant stated:

Participant 02: “*Oh, I think that’s huge because that’s such a stressful time and there are always things that arise that don’t come up in everyday life. I think it would be incredibly helpful to be able to continue that while you’re inpatient. Sure. That’s a huge plus.”*

##### *CF*-specific tailoring of program content

The theme of *CF-Specific Tailoring of Program Content* encompassed three subthemes: (1) *Modifications to CBT Skill Application in CF*, (2) *Overall Impression of the Program,* and (3) *Impressions of Proposed CF-Specific Examples.*

Feedback regarding overall impressions of the program was positive and emphasized the value of a CF-specific mental health intervention and greater access to evidence-based mental health care (Table 6). Participant 06 stated:

“*It’s an aspect of CF that has been neglected over the years,”* and another (Participant 01) said, *“Where have you been all my life?… It’s so fundamentally important.… Mental health is a real big issue.”* A 3rd participant (07) stated, “*I think it’s very beneficial for a large majority of CF patients.”*

Examples of how CF-specific content could be integrated into the intervention were provided to participants for feedback. This included common negative thought patterns (e.g., worry and self-critical) for a session identifying maladaptive thoughts and building adaptive thinking skills (e.g., comparing self unfairly to others who have CF or peers who do not have CF), common anxiety-provoking situations for a session on developing an anxiety-management coping plan (e.g., worrying that others will have a negative reaction to coughing in public), and potential health-related goal areas for a session on individualized goal-setting and problem-solving (e.g., completing a specific CF treatment more often or more consistently, or communicating better with their CF team about their needs). Participants were uniformly positive about these, found them relatable, and provided additional CF-specific examples that were subsequently incorporated into the CF-CBT program materials.

Participants were asked directly how each component of CBT skill development included in the proposed intervention should be modified to make it more relevant or accessible for pwCF. After describing the CBT skill, each participant was asked about CF-specific barriers to skill practice and suggested modifications (e.g., “Do you see any ways to tailor this topic so that it can be made especially useful for individuals with CF?”). Participants noted CF-specific barriers and suggested modifications for teaching relaxation skills or creating behavioral activation plans to prevent or relieve depressive symptoms (See Table 6 for exemplar quotes). For example, while diaphragmatic breathing is a common relaxation technique that is taught in CBT-based programs, several participants commented that relaxed breathing may be particularly challenging for pwCF, noting experiences in which attempts to practice deep breathing induced coughing fits or a focus on breathing increased anxiety. Participants provided strategies that circumvented barriers to practicing relaxed breathing (e.g., sitting up instead of laying down, focusing on the rhythm of breathing rather than aiming for a specific length of exhalation) and also suggested other types of relaxation strategies, such as guided imagery, may be less aversive and more helpful for some pwCF. When asked about potential barriers to behavioral activation planning, participants identified barriers related to symptom burden that were further coded/categorized as *Mental Health*, *Physical*, and *Social Considerations*. *Mental Health Considerations* included low motivation to be active or social when feeling stressed, anxious or in a depressed mood. These are typical concerns that need to be addressed when helping patients develop a behavioral activation plan. *Physical* and *Social Considerations* that were noted, however, were unique to the specific context of living with a chronic illness. *Physical Considerations* included not feeling well, fatigue, pain, and mobility limitations, shortness of breath, and treatment burden. Barriers categorized as *Social Considerations* involved concerns about infection control in social/public spaces and social anxiety about coughing in public. Participants also provided suggestions for CF-specific modifications to address these barriers; including setting reasonable goals, pacing activity, and reaching out to friends virtually or through text when in-person contact was limited. See Table 6 for quotes illustrating these identified barriers and suggested modifications to address barriers.

##### Additional recommended topics

Finally, a fourth theme within the major topic areas of Program Development Feedback was *Additional Recommended Topics*. Topics in this theme were categorized as *Content/Context-Oriented* and *Process-Oriented*. Under the heading of *Content/Context*, participants noted that the developers should consider including biological factors that might affect anxiety and mood (e.g., menopause, medication side effects, impact of poor sleep, or physical fatigue related to coughing). Developmental and disease-severity contexts were also discussed as important considerations. Participants noted that there would be different challenges for individuals with CF depending on age, spoke to the importance of addressing anxiety about disease progression and experience of advanced disease, and suggested the need for a developmental adaptation for adolescents with CF. Participants also suggested content on mindfulness, addressing financial stress, survivor’s guilt, and relationships/communication, as well as comorbidities, such as substance use disorder. Process-oriented suggestions included ensuring that the written material was at an appropriate reading level, and the need to emphasize a supportive and flexible approach that normalizes and destigmatizes mental health concerns in CF and that was customizable to the situations and needs of individuals.

## Discussion

Interviews were conducted with key stakeholders to inform decisions about how to tailor the design and implementation of a novel CBT-based intervention to prevent and treat depression and anxiety for adults with CF. Themes highlighted salient stressors for pwCF, especially *Treatment Burden*, *Illness Uncertainty*, and *Financial/Insurance Stress*. *Hospitalizations*, *Self-Perception/Social Comparison*, *Disease Progression*, and *Fear of Death/Dying* were also frequently identified stressors. The identification of *Treatment Burden* as a significant stressor is consistent with prior research (Sawicki, et al., 2013). The daily treatment regimen in CF is estimated to take 2–3 h per day, with treatment complexity and time commitment highest for adults with CF (Sawicki, et al., 2013; Cameron et al., 2022). Illness uncertainty, which has been defined as a complex experience in which it is difficult to find meaning in illness-related events and outcomes are perceived as unpredictable (Mishel, 1983), has been linked with psychological distress and coping in children and adults with chronic illness (Wright et al., 2009; Tackett et al., 2016; Szulczewski et al., 2017). The identification of illness uncertainty as a key stressor in the experience of living with CF further validates the need to modify existing CBT interventions to help patients cope with circumstances in which there is unpredictability in the course of their disease and it is difficult to make plans for the future. As such, the CF-CBT sessions devoted to teaching cognitive coping strategies have been modified from existing CBT protocols that focus on challenging unrealistic or exaggerated thought patterns to emphasizing balanced and flexible thinking and self-compassion. In addition, a session devoted to anxiety management highlights coping skills to manage anxiety about current and future health. As noted above, a comprehensive list of CF-related stressors identified by patients with CF is included in the initial CF-CBT session to decrease stigma about discussing the many concerns that arise. Personally salient stressors that are identified by patients in the initial session can then be targeted in subsequent sessions during which CBT-based coping skills are learned and practiced.

A second major theme identified as part of coping with CF was the interaction of physical symptoms and emotional distress, a topic not often discussed in routine CF care but one that emerged throughout the patient interviews. The negative emotional impact of experiencing a CF exacerbation was highlighted, as was the way in which emotional distress can have both a direct negative impact on physical functioning and an indirect negative impact through disruptions in the ability to sustain daily CF care. Disruptions to sustaining daily care and worsening physical symptoms then become more distressing. This is a foundational theme of the CF-CBT program: helping patients increase their self-awareness of these connections in their own lives, self-advocate for holistic care, and educating CF interventionists in how to better understand and assess these connections with pwCF.

*Resilience*, which is often defined as the capacity for positive adaptation in the face of adversity (Connor and Davidson, 2003), was identified as a third major theme. While pwCF are at higher risk for depression and anxiety than the general population (Quittner et al., 2014), they have also been shown to report higher rates of resilience (Mitmansgruber et al., 2016). Participants in this study discussed the coping strategies they used in the context of illness and hospitalization and identified adaptive thinking patterns that helped them maintain motivation to sustain daily CF care. As such, the CF-CBT program was developed to teach coping strategies to build on strategies already being used by pwCF. Patient-identified coping strategies and examples of adaptive thinking patterns were incorporated throughout the CF-CBT workbook with direct quotes from these interviews, included with participant consent. While CF-CBT is conducted with patients individually, the quotes that appear throughout the workbook may enhance the impact of the intervention by both normalizing the experience of coping with CF and offering a form of peer support.

The second major topic area of the semi-structured interview was patient perceptions of the proposed intervention. This feedback was critical to ensuring the acceptability of program structure, delivery, and CF-specific content. This feedback indicated a high degree of acceptability, and both positive and constructive feedback informed the final design of the intervention. CF-specific modifications, suggested by patients, were incorporated throughout to address potential barriers and facilitate CBT skills practice. Components of the CF-CBT treatment approach, highlighting modifications that were made to tailor the intervention for adults with CF, are summarized in Table 7.

**Table 7:** CF-CBT sessions: summary of goals and CF-specific modifications.

CF-CBT session	Summary of session goals	CF-specific modifications
Session 1: Introduction to CBT	Identify stressors and increase awareness of one’s response to stress Learn about the CBT model and its application to coping with CF Learn a mindfulness strategy to increase awareness of daily moments of joy	Comprehensive list of CF-related stressors included Awareness of the connection between emotional and physical health in CF is emphasized
Session 2: Relaxation Skills	Practice relaxation skills, including guided imagery and progressive muscle relaxation	Two options offered and neither are breathing techniques Discuss how relaxation skills can be applied to manage medical and procedural anxiety
Session 3: Depression in CF: What Helps?	Learn about depression and its impact in CF Develop a personalized behavioral activation plan	Psychoeducation about the increased risk for depression and health consequences of untreated depression in CF Planning includes problem-solving back-up options and adaptive pacing of activities to manage interference of CF symptom and treatment burden Create a specific plan for behavioral activation that could be especially helpful in times of illness or hospitalization
Session 4: Adaptive Thinking Skills	Increase awareness of thinking patterns that contribute to depression and anxiety	Learn about maladaptive thinking patterns that relate to coping with CF Address thinking patterns that decrease motivation to sustain daily CF care
Session 5: Adaptive Thinking Skills, Part 2	Practice strategies for developing new thought patterns that are more adaptive	Balanced and flexible thinking and self-compassion is emphasized Incorporate mindful “letting go” of maladaptive thoughts to manage CF-related stress
Session 6: Taking Charge of My Health	Learn goal setting and problem-solving skills and apply toward a patient-identified health-related goal	CF-specific examples include goals of improving consistency of daily CF care, communication with the CF team, and increasing comfort with disclosure about CF when needed
Session 7: Anxiety in CF: What Helps?	Learn about anxiety and strategies for coping Develop a personalized anxiety management plan	Psychoeducation about the increased risk for anxiety and health consequences of untreated anxiety in CF The ways that anxiety can interfere with self-care are addressed Anxiety about current and future health and coping with uncertainty is discussed
Session 8: Maintaining Positive Changes	Create an individualized follow-up plan that includes continued practice of preferred coping skills to face future challenges	Plan is coordinated within CF-team based care, allowing for coproduction and flexible, longitudinal follow up

Patient community engagement was invaluable to adapting CBT-based protocols in the development of CF-CBT to better address the following goals: (1) normalize and destigmatize distress related to managing a progressive chronic illness, (2) emphasize the intersection of physical and emotional health in ways that are specific to the experience of CF, particularly the impact of depression and anxiety on sustaining daily CF self-care, (3) address health-related anxiety, (4) problem-solve CF-specific barriers to skill implementation, and (5) address CF-specific barriers to mental health care (e.g., existing burden of medical/daily self-care).

There were limitations to this qualitative study. While a wide range of age and disease severity was represented among participants, gathering additional feedback from a more broadly diverse sample, especially with regard to race and ethnicity, would improve the intervention’s relevance for a diverse population of pwCF. CF-CBT was developed prior to the advent of highly effective modulator therapy in CF and the COVID-19 pandemic. Each of these events individually and interactively had an impact on coping. Additional qualitative work will follow to better understand the impact of these events on pwCF and to update the program materials accordingly.

Although the program will be further refined, results of these qualitative analyses indicate the acceptability of a CF-specific CBT-based mental health intervention for adults with CF that was designed to be integrated into routine CF care. CF-CBT was subsequently piloted, with positive preliminary findings regarding feasibility, acceptability, and effectiveness (Friedman et al., 2022), with additional changes made prior to an ongoing multi-center randomized waitlist-controlled trial. Qualitative results directly informed the content of CF-CBT, increasing its validity and potential to accurately reflect CF-specific experiences and accommodate CF-specific coping needs. Positive participant feedback on acceptability was received for the pilot study confirming the value of including the patient community in intervention development. Given the elevated prevalence of depression and anxiety across chronic illness populations, the approach described here may be replicable for co-developing mental health interventions for chronic illness populations tailored to patient needs. Interventions that target the contextually relevant challenges faced by individuals with a specific chronic condition are more compelling to patients and more effective (Quittner et al., 2019). Customized interventions can decrease stigma and increase patient engagement in evidence-based mental health care.

## Data availability statement

The datasets presented in this article are not readily available because the datasets for this study cannot be made publicly available per the hospital review board policies. Requests to access the datasets should be directed to dfriedman@mgh.harvard.edu.

## Ethics statement

The studies involving human participants were reviewed and approved by Massachusetts General Hospital Institutional Review Board. Written informed consent for participation was not required for this study in accordance with the national legislation and the institutional requirements.

## Author contributions

DF: conceptualization, methodology, data collection/interviewing, data management, formal analyses, writing—original draft preparation, reviewing, editing, visualization, and formatting. MK: conceptualization, methodology, formal analyses, writing—original draft preparation, reviewing, and editing. AQ: conceptualization, methodology, data collection/interviewing, formal analyses, reviewing, and editing. BS: conceptualization, methodology, data collection/interviewing, reviewing, and editing. AG: conceptualization, methodology, reviewing, and editing. All authors contributed to the article and approved the submitted version.

## Funding

The study reported in this manuscript was supported through a 2016 Circle of Care Award from Vertex Pharmaceuticals, Inc., and a Cystic Fibrosis Foundation Therapeutics, Inc. research grant (FRIEDM17A0). The funders were not involved in the study design, collection, analysis, interpretation of data, the writing of this article or the decision to submit it for publication.

## Conflict of interest

DF reports grant support from the Cystic Fibrosis Foundation (CFF), Dutch Cystic Fibrosis Foundation, and Vertex Pharmaceuticals, and travel reimbursement from the CFF. BS reports grant support, personal fees and travel reimbursement from CFF. AQ reports consulting fees from Vertex Pharmaceuticals and Insmed, and grant support from the CFF, American Cochlear Implant Alliance, PCORI, and the NIH. AG reports personal fees and travel reimbursement from Cystic Fibrosis Australia; grant support, personal fees, and travel reimbursement from CFF; grant support from the Dutch Cystic Fibrosis Foundation; travel reimbursement from the European Cystic Fibrosis Society; personal fees from Johns Hopkins University/DKBmed; personal fees from Saudi Pediatric Pulmonology Association; and grant support and personal fees from Vertex Pharmaceuticals. MK reports no commercial or financial relationships that could be construed as a potential conflict of interest.

## Publisher’s note

All claims expressed in this article are solely those of the authors and do not necessarily represent those of their affiliated organizations, or those of the publisher, the editors and the reviewers. Any product that may be evaluated in this article, or claim that may be made by its manufacturer, is not guaranteed or endorsed by the publisher.
